# First report of a phylogenetic analysis of an autochthonous *Plasmodium vivax* strain isolated from a malaria case in East Attica, Greece

**DOI:** 10.1186/1475-2875-12-299

**Published:** 2013-08-29

**Authors:** Anastasios Ioannidis, Chryssoula Nicolaou, Athina Stoupi, Athanasios Kossyvakis, Petros Matsoukas, Melina-Vassiliki Liakata, Emmanouil Magiorkinis, Efthimia Petinaki, Stylianos Chatzipanagiotou

**Affiliations:** 1 Department of Nursing, Faculty of Human Movement and Quality of Life Sciences, University of Peloponnese, Sparta, Greece; 2 Department of Biopathology and Clinical Microbiology, Athens Medical School, Aeginition Hospital, Vass. Sophias av. 72-74, Athens 115 28, Greece; 3 Athens Medical Centre - Peristeri, Peristeri, Greece; 4 Department of Hygiene, Epidemiology and Medical Statistics, Medical School, University of Athens, Athens-Goudi, Greece; 5 Department of Microbiology, Medical School, University of Thessalia, Larissa, Greece

**Keywords:** Malaria, Greece, *Plasmodium vivax*, CSP, MSP-1, Phylogenetic analysis

## Abstract

Malaria has become an emerging infection in Greece, which is the doorstep to Europe for thousands of immigrants. With increasing immigration, cases with evidence of domestic transmission (autochthonous) are being reported. In the present study, an isolate of *Plasmodium vivax* from an autochthonous clinical case was subjected to phylogenetic analysis of the genes encoding the merozoite surface protein 1 (MSP-1) and the circumsporozoite protein (CSP). In the MSP region, the strain was related with strains from Brazil, South Korea, Turkey and Thailand, whereas in the CSP region, with strains from Brazil, Colombia and New Guinea. The present study establishes for the first time in Greece the basis for the creation of a database comprising genotypic and phylogenetic characteristics of *Plasmodium* spp.

## Background

In 1974, Greece was officially declared as “malaria free” and until 2008 only imported cases were notified. However in 2009, with increasing immigration, isolates with evidence of domestic transmission (autochthonous) were reported from Hellenic Center for Disease Control and Prevention [1]. This study presents a recent autochthonous case of *Plasmodium vivax* malaria in a Greek woman from East Attica. The isolated *Plasmodium* strain was phylogenetically analysed in order to establish a database as a tool for further epidemiological monitoring.

## Case presentation

### Clinical data

**Figure 1:**
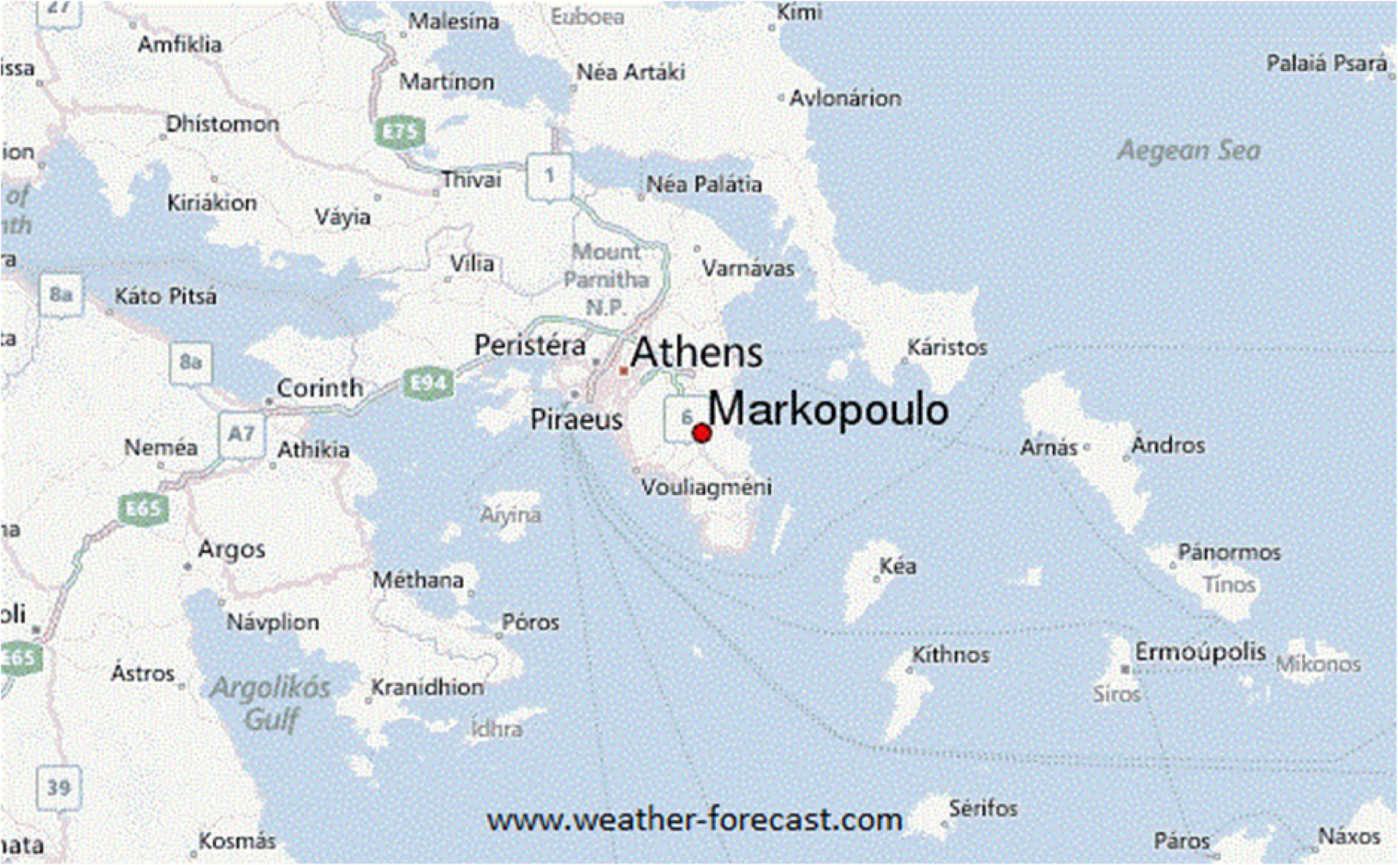
Map of Attica showing the city of Markopoulo where the autochthonous malaria case was isolated.

The patient, a 44 year-old woman, and a permanent resident in Markopoulo, a city in East Attica (Figure 1), was admitted on 11 July, 2012 to the outpatient department of the private hospital Athens Medical Center- Peristeri, with high fever (39°C) for the previous ten days, starting with rigour. There was no history of previous travel abroad, blood transfusion, tissue organ transplantation, intravenous drug abuse or a prolonged febrile illness over the last year. Heart and abdomen examinations were normal. Blood cultures (six) were negative. The haematological examination revealed a progressively aggravating pancytopaenia. The serological viral and bacterial investigations for EBV, CMV, adenovirus, parvovirus, coxsackie, HIV, *Rickettsiae*, *Chlamydia* and spirochetes were negative. Chest computer tomography (CT) was normal. CT of the lower abdomen showed a mild splenomegaly (about 15 cm). Transthoracic echocardiograph, breast ultrasound (required because of the family history reporting breast cancer of her mother and sister) and MRI (including angiography) of the brain were normal and could not justify the fever attacks. PCR for *Leishmania* was negative. Microscopic examination of thick and thin blood smears, performed eight days after admission, was positive for *P. vivax*. The result was also confirmed by PCR, and then several blood samples were sent to special laboratories for further molecular processing.

The patient was successfully treated, initially with atovaquone/proguanil (Malarone®, 1 g atovaquone /400 mg proguanil total daily dose, referring to four tablets as a single dose) for three consecutive days and then for 14 days with primaquine (30 mg daily dose, after examination for G6PDH deficiency).

## Consent

Ethical approval and patient consent was not required for the study and the samples taken were part of the standard patient care.

### Phylogenetic analysis

Phylogenetic analysis of the isolated strain, as well as from *Plasmodium* strains from worldwide composed databases, was performed with respect to the highly polymorphic genes encoding the merozoite surface protein 1 (MSP-1) and the circumsporozoite protein (CSP) [2,3].

MSP-1 is a 200-kDa protein expressed on the surface of the *P. vivax* merozoite, plays a key role during erythrocyte invasion and is a target of host protective immune response [4]. Disruption of the *Plasmodium msp-1* gene has been demonstrated to have a deleterious effect on the parasite growth in experimental animals [5]. MSP-1 is a valuable polymorphic marker, organized into several variable blocks, flanked by ten conserved sections and having a dimorphic pattern [6]. Block 5, the region encompassed by the interspecies conserved block ICB5 and ICB6, shows a dimorphic pattern of sequences that have little homology. These patterns are mainly composed of three major types (Sal-I, Belem, and Recombinant) and their subtypes [2,6]. CSP covers the surface of infectious sporozoites involved in the sporozoite invasion of hepatocytes and has been considered an important vaccine candidate [7]. The central repetitive domain from the CSP varies in sequence and length among *Plasmodium spp*. The classic *P. vivax* VK210 strain has CSP amino sequence that includes a GDRAA/DGQPA repeat [8]. A variant form, VK247, later identified in Thailand, possesses an ANGAGNQPG amino acid repeat within the amino acid tandem repeat region [9]. All CSP variant genotypes have a worldwide distribution [10,11]. This polymorphic marker was useful for genetic epidemiological surveys where *P. vivax* is endemic.

For phylogenetic analysis, the *P. vivax* genes for MSP-1 and the CSP were first PCR amplified and sequenced. DNA was extracted from 200 μl of patient blood using the QIAamp DNA Blood Mini kit (Qiagen, Hilden, Germany) according to the manufacturer’s instructions. Primer sequences for the amplification of the DNA fragment encompassing the *csp* gene and the ICB5-ICB6 region of the *msp-1* gene were adopted from previous reports [2]. Optimization of the above PCR assays for the primers concentration, as well as for the annealing temperature, was performed using Go Taq® Hot Start Colorless Master Mix, 2× (Promega, Madison, WI, USA) on a Eppendorf Mastercycler gradient thermal cycler.

The PCR resulting amplicons were purified utilizing the QIAquick PCR purification kit (Qiagen, Hilden, Germany) and the MinEluteTM gel extraction kit (Qiagen, Hilden, Germany) and sequenced in both directions using the GenomeLab DTCS-quick start sequencing kit (Beckman Coulter, Brea, CA, USA) on a CEQTM 8000 genetic analyzer (Beckman Coulter, USA).

For the genetic comparison and the phylogenetic analysis, the *P. vivax* gene sequences amplified from the malaria patient’s blood specimen were compared with previously published *P. vivax msp-1* and *csp* gene sequences on GenBank of National Center for Biotechnology Information. Sequences from the patient have been deposited in the GenBank database under the accession numbers KC682100 for *msp-1* and KC896384 for *csp* genes. Multiple sequence alignment of all the gene sequences and loci was performed with the algorithm Clustal W using the MEGA (Molecular Evolutionary Genetics Analysis) 5.05 software. Alignments were manually edited and insertions/deletions in coding regions were determined from multiple alignments of amino acid sequences to maintain the reading frame. Genetic distances between the sequences were calculated using the Tamura-Nei model [12,13]. Phylogenetic trees were constructed using the neighbour-joining method and their reliability was tested by bootstrapping analysis (1,000 replicates). One cluster was considered significant if it was present in more than 75% of the permuted trees.

## Conclusions

**Figure 2:**
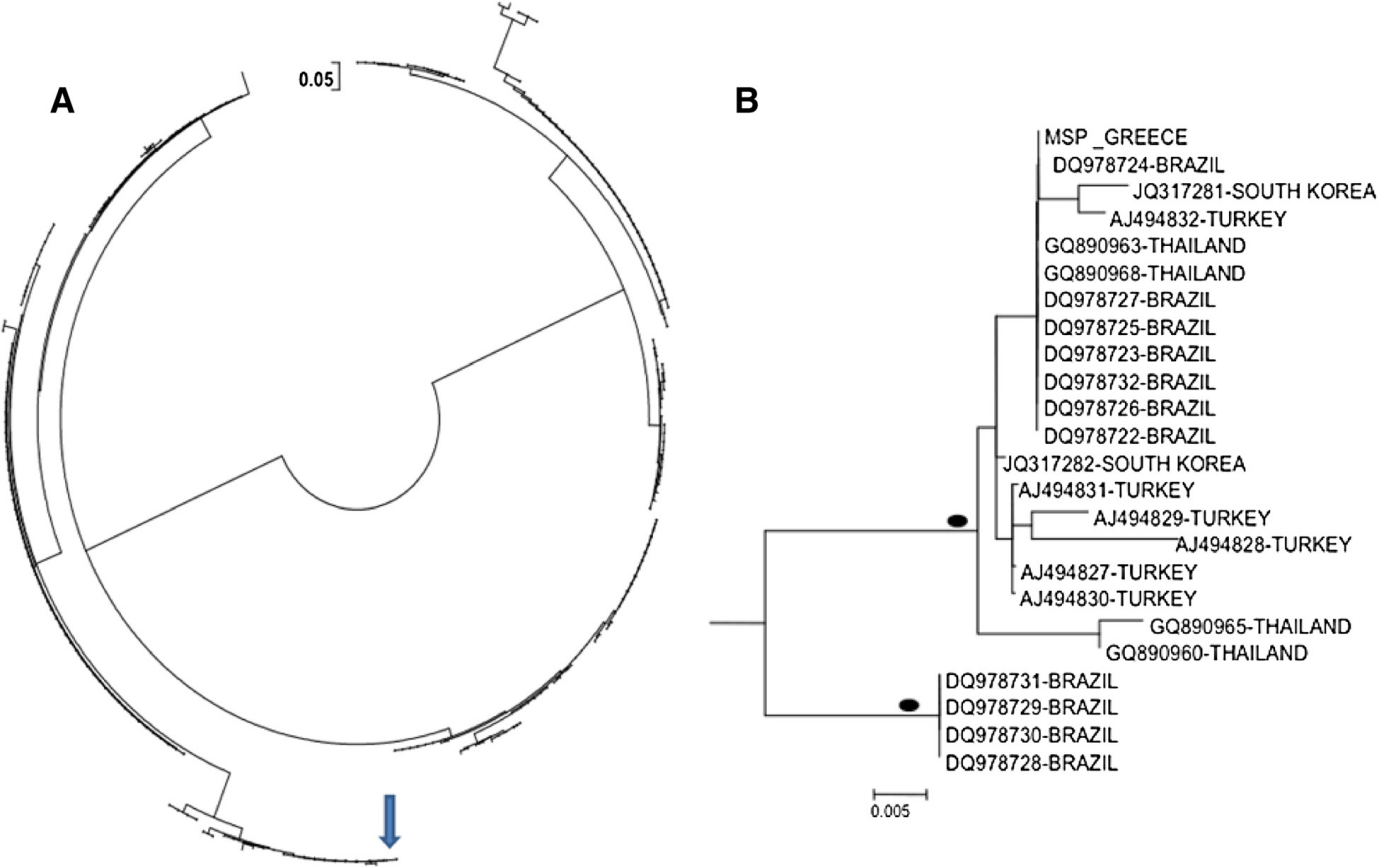
**Neighbour-joining tree of *Plasmodium vivax *merozoite surface protein-1. A.** MSP-1 gene sequences from the strain obtained from Greece, as well as from the GenBank. **B.** Subtree including the Greek strain and depicting its phylogenetic relationship with other strains. Bullets represent >75% bootstrap value (1,000 replicates).

**Figure 3:**
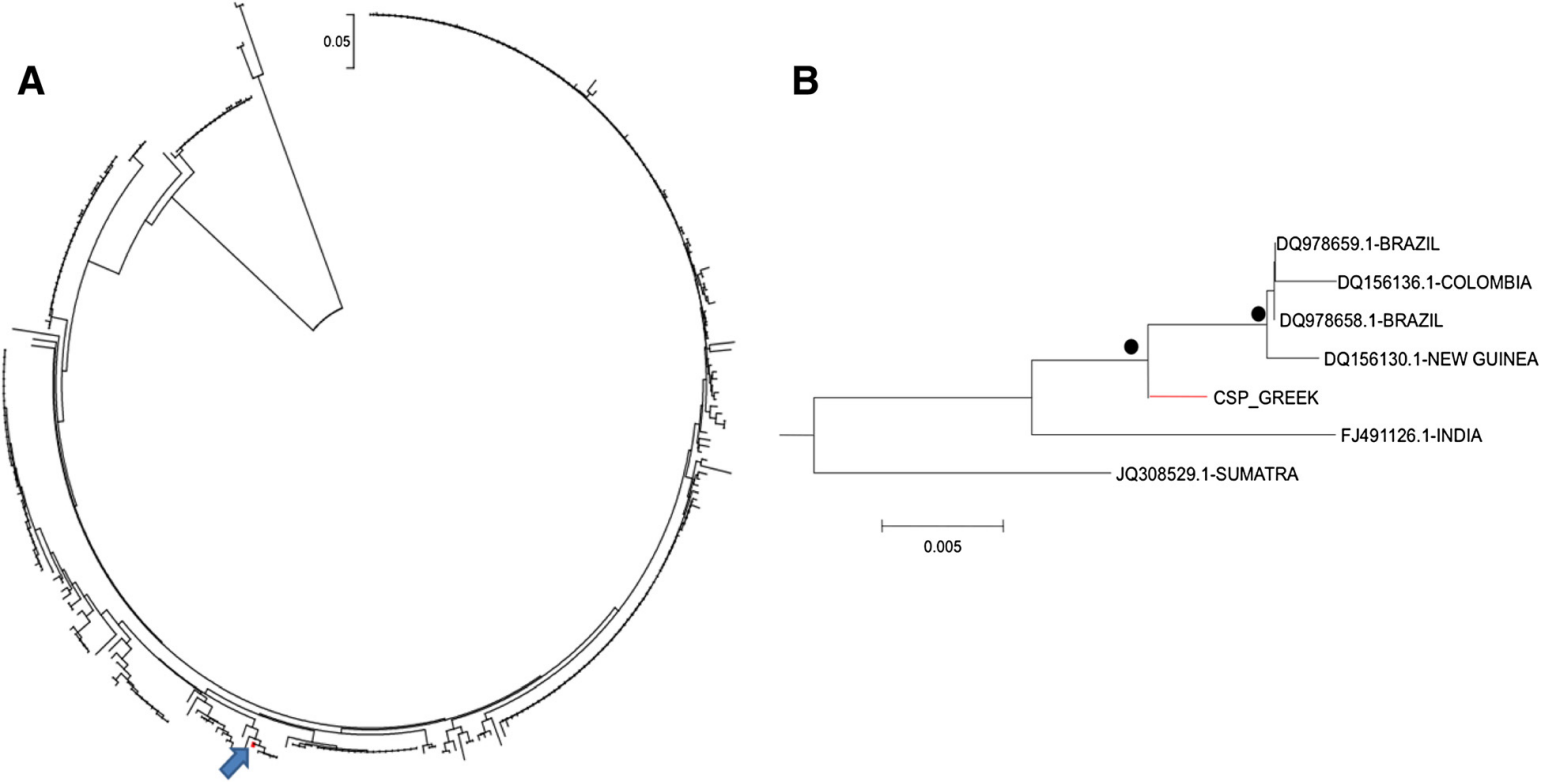
**Neighbour-joining tree of *Plasmodium vivax *circumsporozoite protein. A.** CSP gene sequences from the strain obtained from Greece, as well as from the GenBank. **B.** Subtree including the Greek strain and depicting its phylogenetic relationship with other strains. Bullets represent >75% bootstrap value (1,000 replicates).

Phylogenetic analysis in the MSP region showed that the investigated strain was related with strains from Brazil, South Korea, Turkey and Thailand (Figure 2A and B), whereas in the CSP region it clustered with strains from Brazil, Colombia and New Guinea (Figure 3A and B).

Taking into consideration previously published studies [2,6,8-11,14], the amino acid sequence was in the MSP and CSP regions with reference isolates. Regarding the MSP region, the strain belonged to the Belem type, whereas with respect to the CSP region, to the VK210 type (alignment not shown). The obvious discrepancy between the results of the phylogenetic analysis in the MSP and CSP regions is due to the absence of adequate *Plasmodium* sequence data at the GenBank database and to the absence of adequate number of paired sequences from the same strain [14]. However, using *msp1* and *csp* as genetic markers, the close proximity of the strain in both regions with strains from South America in both trees (Brazil, Colombia) denotes a possible origin of the *Plasmodium* strain from South America. Further studies, including isolates of autochthonous cases, will be required in order to obtain a clearer picture of *Plasmodium* diversity in Greece.

The present study establishes, for the first time in Greece the basis for the creation of a database comprising genotypic and phylogenetic characteristics of *Plasmodium* spp. Malaria has become an emerging infection in Greece, which is the doorstep to Europe for thousands of immigrants. Therefore clinicians should be aware of considering malaria early in their differential diagnosis. The enrichment in the future with new strains of imported and autochthonous cases will be an invaluable tool for the epidemiological monitoring of malaria in Europe.

## Abbreviations

MSP-1: Merozoite surface protein 1; CSP: Circumsporozoite protein; EBV: Epstein-Barr Virus; CMV: Cytomegalovirus; MEGA: Molecular evolutionary genetics analysis.

## Competing interests

The authors have declared that there are no financial or non-financial competing interests.

## Authors’ contributions

SC designed and wrote the article. AS, PM, MVL and CN diagnosed and treated the patient and collected all the clinical and diagnostic laboratory data. AI, AK, EM and EP designed, performed and assessed the molecular and phylogenetic analysis. All authors read and approved the final version.
